# Cell–cell signalling in sexual chemotaxis: a basis for gametic differentiation, mating types and sexes

**DOI:** 10.1098/rsif.2015.0342

**Published:** 2015-08-06

**Authors:** Zena Hadjivasiliou, Yoh Iwasa, Andrew Pomiankowski

**Affiliations:** 1 Centre for Mathematics, Physics and Engineering in the Life Sciences and Experimental Biology (CoMPLEX), University College London, Gower Street, London WC1E 6BT, UK; 2 Department of Genetics, Evolution and Environment, University College London, Gower Street, London WC1E 6BT, UK; 3 Department of Biology, Faculty of Sciences, Kyushu University, Fukuoka 812–8581, Japan

**Keywords:** signalling, chemotaxis, mating types, sexes

## Abstract

While sex requires two parents, there is no obvious need for them to be differentiated into distinct mating types or sexes. Yet this is the predominate state of nature. Here, we argue that mating types could play a decisive role because they prevent the apparent inevitability of self-stimulation during sexual signalling. We rigorously assess this hypothesis by developing a model for signaller–detector dynamics based on chemical diffusion, chemotaxis and cell movement. Our model examines the conditions under which chemotaxis improves partner finding. Varying parameter values within ranges typical of protists and their environments, we show that simultaneous secretion and detection of a single chemoattractant can cause a multifold movement impediment and severely hinder mate finding. Mutually exclusive roles result in faster pair formation, even when cells conferring the same roles cannot pair up. This arrangement also allows the separate mating types to optimize their signalling or detecting roles, which is effectively impossible for cells that are both secretors and detectors. Our findings suggest that asymmetric roles in sexual chemotaxis (and possibly other forms of sexual signalling) are crucial, even without morphological differences, and may underlie the evolution of gametic differentiation among both mating types and sexes.

## 1. Introduction

The evolution and persistence of different sexes and mating types has received remarkably little attention compared with that lavished on the value of sexual reproduction [1]. The difference between sexes manifests itself in morphological and functional asymmetry at the gametic and organism level. This is most obviously seen among multicellular organisms, but extends back to unicellular eukaryotic forms. However, many protists retain morphologically identical gametes (isogamy), typically associated with little dimorphism at the organismal or vegetative stage. Despite this apparent similarity, only gametes of different mating types can fuse, with unions between gametes of the same mating type being very rare. While sex requires two gametes, there is no obvious necessity that these are from different mating types, particularly without any seeming morphological or behavioural differences. So the forces leading to incompatible mating types is a distinct and fundamental question to address in understanding the origins of gametic differentiation.

A popular explanation for the evolution of mating types relates to organelle inheritance. According to this view, mating types evolved because two different gamete types can enforce uniparental inheritance of cytoplasmic symbionts in which one mating type passes on its cytoplasm while the other does not. Such mechanisms are present in many isogamous protists and avoid cytoplasmic mixing from two parents, restricting the spread of mutations and parasitic elements or preventing conflict between unrelated organelles [2,3]. Considerable theoretical effort has been expended on understanding this hypothesis, and initially supported the idea [4–9]. However, recent work shows that the relative advantage of uniparental inheritance declines within a population in a frequency-dependent manner, casting significant doubts on the potential of this theory to explain the evolution of mating types [10]. In addition, as pointed out by others, many isogamous protists that have bidirectional cytoplasmic inheritance or no cytoplasmic mixing during sex maintain mating types and do not fit this hypothesis [11,12].

Another dominant hypothesis proposes that mating types are important because they promote outbreeding and prevent same clone fusions [13]. This hypothesis has a strong appeal, as inbreeding can indeed be detrimental in many higher animals and plants [14], and high levels of inbreeding are harmful in some protists [15]. However, many protists that have a diploid vegetative stage are heterozygous for mating type loci in their adult state. They produce equal numbers of gametes of the two different mating types which are compatible with one another [11,16,17]. This begs the question as to why these organisms maintain mating types, and why mating types are not determined at the diploid level so as to prevent inbreeding. Furthermore, many ciliates and fungi have evolved elaborate mechanisms for mating type switching which once again means that they can undergo selfing or same clone mating [18,19]. These mating types are important as they code for interactions that promote gamete formation, partner finding, recognition and fusion. The avoidance of inbreeding is much more powerfully induced by the presence of self-incompatibility alleles that hinder fusion of anisogametes (egg and sperm) produced by the same individual or clone. Although these are common in higher plants, they are rarely found in protists [20–22]. These considerations suggest that inbreeding avoidance is not the crucial force maintaining two mating types in protists.

A further hypothesis was proposed by Hoekstra in a series of papers [23–25]. This model suggests that mating types are determined by the biophysical properties of the molecular system underlying gamete interactions. According to this hypothesis, gamete recognition and pairing are more efficient when gametes produce a recognition/attraction molecule or its receptor in a mating-type-specific manner. Mating-type-specific production of ligand/pheromones and their receptors has been documented in many isogamous protists, including examples from fungi [26], algae [27] and ciliates [28]. However, the asymmetric signalling idea has been omitted or dismissed in recent reviews on the origins and significance of mating types [11,12,29]. This neglect in part reflects the popular assumption that asymmetric interactions exist to impose opposite mating-type fusions for reasons unrelated to the signalling interaction itself (e.g. control of organelle inheritance; inbreeding avoidance). But in addition, a theoretical analysis of the biophysical properties of gamete signalling within particular environments is lacking. Without this it is hard to know under what conditions asymmetric signalling might be favoured, how this relates back to real organisms, and whether its potential benefits are strong enough to hold a role in the evolution of gamete differentiation.

**Figure 1.**
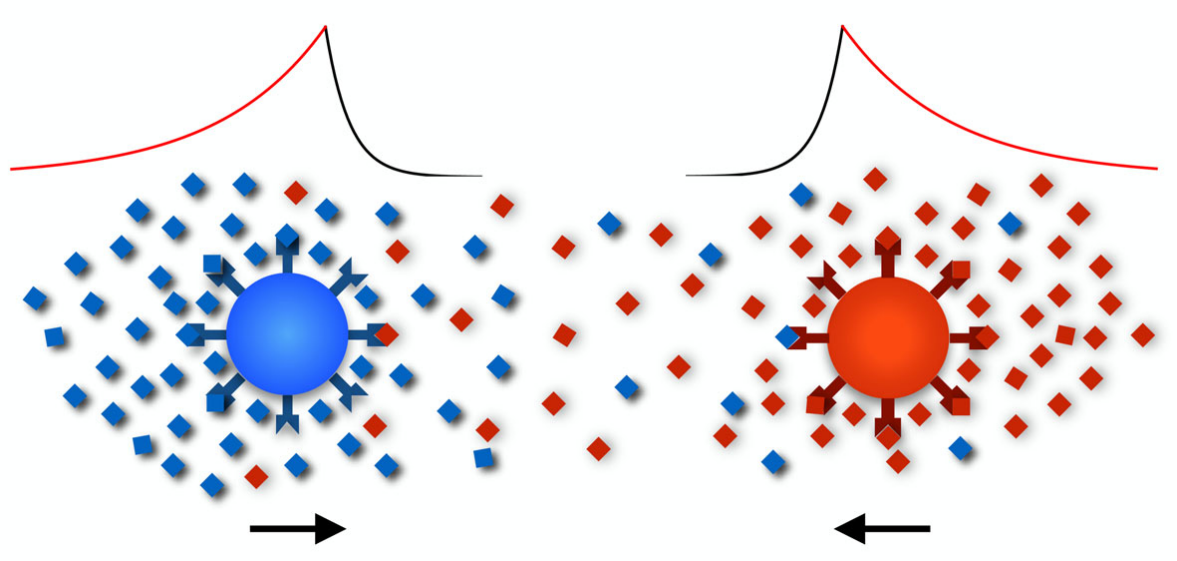
Chemical concentration around moving secretors. Cells secrete a diffusible chemical (red and blue diamonds) that binds to membrane receptors thereby inducing a chemotactic signal. Molecules secreted by the cell can cause two problems. First, they bind to the cell's own receptors causing saturation and interference with signals from remote partners whose molecules are always at relatively low concentration because of diffusion (the red cell has most of its receptors occupied by its own pheromone). Second, owing to cell movement, secretion causes a tail of high concentration behind the moving cell. It follows that receptor occupancy is higher behind the moving cells, prompting the cell to repeatedly reverse direction (the blue cell's receptors are occupied mainly at its rear).

Secreting and detecting the same cue can be problematic when a quick and accurate response to an external signal is desirable, particularly in chemotaxis where cells continuously respond to chemical fields by adjusting their movement [30,31]. This is largely intuitive as the local concentration of the chemoattractant because of a cell's own signal will always be higher than that of a remote signaller, triggering the cell's own receptors and impairing the perception and clear response to an external signal (figure 1). Furthermore, secretion during movement causes a tail of high concentration behind the moving cell because of diffusion and accumulation of chemical molecules. Such self-induced asymmetry alters the net local concentration, reducing the cell's ability to respond appropriately to external signals or worse, prompting the cell to reverse its direction of movement (figure 1; also see [31]). The significance of these considerations depends on the environment (medium, cell density, chemical diffusivity), the physiology of the cells (cell size, speed) and the purpose that chemotaxis serves (aggregation, dispersal, nutrient finding, pair formation).

Here we revisit the idea that mating types and mating-type-specific molecular interactions can improve partner attraction and mating by quantifying the effects of simultaneous chemical secretion and detection on the capacity of gametes to form pairs. Our study expands our understanding of mating dynamics from an ecological and physiological viewpoint. This allows us to relate our results back to protists, their physicality, life cycles and environments. In doing so, we also provide an explicit quantitative analysis of chemotaxis inhibition by self-secretion under different conditions. Although Hoekstra's initial theory dealt with both recognition (surface bound) and attraction (diffusible) signals, we only investigate the latter. In the Discussion, we consider how our results relate to non-chemotactic diffusible signals, and point toward the study of signalling interactions that are surface bound.

## 2. Methods and model outline

We construct a two-dimensional model of individual cell movement and chemical diffusion. The model captures general principles of unicellular protist movements and responses to chemical gradients, but does not consider details of the actual propulsion mechanism.

### 2.1. Chemical field

Cells contribute to a chemical gradient by secreting a diffusible pheromone. The time evolution of the chemical field *C*(**x**, *t*) is thus governed by a diffusion equation with a source term that depends upon the secreting cells' trajectories,2.1
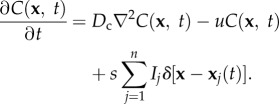
The above equation is the classic diffusion equation with a degradation term, and a source contribution that depends on the trajectory of secreting cells [31,32]. Here, *D*_c_ is the chemical diffusivity of the pheromone in the medium, *u* is the chemical degradation rate, *s* is the secretion rate per cell and *n* is the number of cells present. The indicator factor *I_j_* is equal to 1 if the *j*th cell secretes the pheromone and 0 otherwise, the vector **x***_j_*(*t*) is the trajectory of the *j*th cell from time 0 to time *t*, and *δ*(**y**) is the Dirac delta function and is equal to 1 if **y** = 0, and 0 otherwise.

Assuming that cells start to produce pheromone at time *t* = 0, with the help of Green functions, we obtain the solution of equation (2.1) which is given by2.2
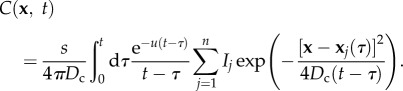


Similarly, the gradient of the chemical concentration is given by2.3
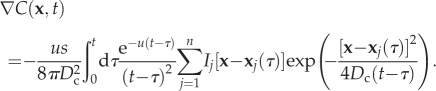
Numerical integration of equations (2.2) and (2.3) is used to obtain the chemical concentration and gradient at a cell's position at time *t* throughout our analysis, respectively (see the electronic supplementary material for detailed derivation and numerical methods).

### 2.2. Cell movement

We simulate cell movement in time steps of *μ* = 0.1 s. Several studies indicate that eukaryotic cells switch between periods of nearly straight-line swimming and relatively swift reorientations [33,34]. Here, we model this general behaviour by assuming that cells move in a direction for a period determined by a persistence parameter, *p*, equal to the probability that a cell maintains its orientation at a given time step.

In the absence of chemical receptors or a chemical gradient, cells move non-chemotactically. In this case, the updated cell orientation is an arbitrary angle *θ* drawn from a Unif [0, 2*π*] distribution. It follows that the cell's new position will be given by (*x*_0_ + *l* cos*θ*, *y*_0_ + *l* sin *θ*), where (*x*_0_, *y*_0_) is the cell's position prior to the reorientation and *l* is the length of the step randomly chosen from a Unif [0, 2*vμ*] distribution. Under this formulation the average length of the step taken by a cell in time *μ* is equal to *vμ*, where *v* is the average cell speed.

**Figure 2.**
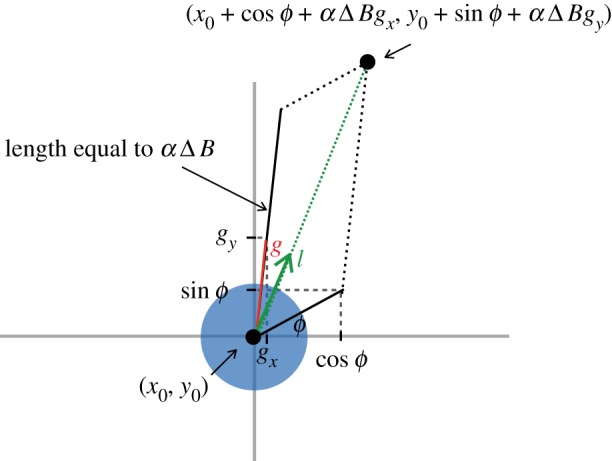
Chemotactic cells change their direction according to the chemical gradient. The vector ***g*** shown in red is a unit vector along the direction of the gradient. The cell updates its position by taking a step of length *l* along the direction of the dotted green vector which is the sum of a unit vector along a random direction and a magnified vector along the direction of the gradient. The greater this magnification (determined by *α*Δ*B*), the closer is the direction the cell moves in to the direction of the gradient. *l* is chosen from a uniform distribution on [0, 2*vμ*].

In the presence of a chemical gradient, cells that possess surface receptors sensitive to the pheromone respond by becoming polarized along the chemical gradient (determined by solving equation (2.3) at the centre of the detecting cell). This defines the cell's front and rear along the gradient (figure 2). Cells move in the direction of the gradient with fidelity proportional to the difference in receptor occupancy across their polarized ends (computed using equation (2.2) at the respective coordinates). Purely spatial gradient sensing via saturable membrane receptors is common among eukaryotic cells [35,36]. We model receptor binding using Hill functions [35–37], so that the fraction of occupied receptors at any point on the cell's membrane obeys the equation *B* = *C*/(*C* + *K*_d_), where *K*_d_ is the dissociation constant of the pertinent receptors and *C* = *C*(**x**, *t*). We assume that polarization along the gradient depends linearly on the difference in receptor occupancy across the cells' polarized ends, Δ*B*, where
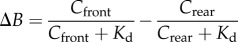
and *C*_front_ and *C*_rear_ are the concentrations at the front and rear of the polarized cell, respectively.

We define *α* to be the strength of a cell's response to the chemical gradient—the larger the value of *α*, the more precise the alignment of the cell to the chemical gradient. This is effectively a measure of the amplification that occurs within the cell, inducing a response to the external chemical signal. It follows that the cell position at time *t* + *μ* is a step of length *l* along the direction given by the vector (*x*_0_ + *x*_1_ + *α*Δ*B g_x_*, *y*_0_ + *y_1_* + *α*Δ*B g_y_*) (figure 2). Here *l* is chosen randomly from a Unif [0, 2*vμ*] distribution, (*x*_0_, *y*_0_) is the position of the cell at time *t*, (*x*_1_, *y*_1_) = (cos*ϕ*, sin*ϕ*) is a random unit vector with *ϕ* sampled from a Unif [0, 2*π*], and (*g_x_*, *g_y_*) is the unit vector in the direction of the gradient found using equation (2.3). The higher the coefficient *α*Δ*B* is, the closer the cell's direction is to the gradient.

For all types of cells we also add an error term so that small fluctuations in cell orientation are allowed even if the cell in question does not update its polarity and orientation (details in electronic supplementary material). This is effectively an implementation of extrinsic noise. The terms and parameters of our model are summarized in table 1.

**Table 1.** Key terms and definitions.

*d*	cell diameter
*v*	cell speed
*p*	cell movement persistence
*C*(**x**, *t*)	chemical concentration at **x** at time *t*
*D*_c_	diffusion coefficient
*u*	chemical degradation
*s*	secretion rate per cell
*B*	proportion of occupied receptors
*K*_d_	receptor dissociation constant
*D*	diffusion coefficient
*α*	chemotactic constant
NC	non-chemotactic cells
SD	secrete-and-detect cells
S + D	secrete-only and detect-only cells

## 3. Results

We model the sexual phase of the protist life cycle when vegetative cells produce isogametes. An environment is simulated where many cells are present, searching for a partner. The relative advantage of sexual chemotaxis is assessed by contrasting three cases: (i) all cells in the population can mate with one another and are non-chemotactic (NC), (ii) all cells in the population can mate with one another and are both signallers and detectors (SD), and (iii) half of the cells are signallers (S) and half are detectors (D). In the latter scenario, cells from the same groups may not fuse, i.e. we assume mating types with mating-type-specific roles in chemotactic signalling. Although this limitation may seem strict, it serves to quantify trade-offs between asymmetric chemotaxis and mating incompatibility. We focus our analysis on ecological parameters pertinent to small protists. Table 2 provides a range of values for the cell speed and diffusion coefficients that span known or anticipated values in unicellular eukaryotes.

**Table 2.** Indicative values for the diffusion coefficient (*D*), cell speed (*v*) and cell diameter (*d*) in protists. References are shown in square brackets.

diffusion coefficient		cell speed		cell size	
molecule	*D*_c_ (cm^2^ s^−1^)	cell type	*v* (μm s^−1^)	cell type	*d* (μm)
small molecule	1–1.4 × 10^−5^ [38]	flagellated cells	20–200 [39]	*Chlamydomonas reinhardtii*	10 [40]
small protein	approximately 4 × 10^−6^ [38]	ciliates	150–2000 [39]	amoebas	20–500 [41]
cAMP	approximately 4 × 10^−6^ [42]	amoebas	<5 [39]	cercomonads	4–65 [43]
yeast *α*-factor	3.2 × 10^−6^ [44]	*C. reinhardtii*	approximately 100 [45]	ciliates	80–200 [46]
glycoproteins^a^	10–0.1 × 10^−5^ [38,47]	*Paramecium tetraurelia*	140–470 [48]	diatoms	2–200 [49]

^a^Common pheromones of protists are glycoproteins.

A number of the key parameters in the model were varied to quantify their role in the behaviour of the three types of cell movement. The results are organized into four sections considering movement persistence, cell speed and pheromone diffusion, the chemotactic constant, and finally cell diameter. In all of these, we consider assemblies of cells (i.e. *n* > 2) with an initial cell density *ρ*_0_ = 5.1 × 10^6^ cells m^−2^. We used periodic boundary conditions which means the cell density is important, not the absolute number of cells (see Methods and model outline and the electronic supplementary material for proof of convergence). This value is equivalent to an intermediate level of cell density as measured in a range of microbial species [50]. A sensitivity analysis was carried out to illustrate the robustness of our findings (electronic supplementary material, figures S7, S9, S11–S13).

We use the half-life (*h*) as a measure of the speed of pair formation, the time until 50% of the initial population has found a partner (two cells mate once in physical contact). Large *h* indicates poor mate-finding performance. *h*_NC_, *h*_SD_ and *h*_S+D_ denote the half-life when all cells are NC, all cells are SD and half the cells are S + D, respectively. The half-life is a good measure for comparison as it captures the rate at which cells form pairs. Metrics that measure the distribution of mating times as opposed to a rate, such as the mean, are less appropriate as they can be heavily skewed by the large times it takes the last remaining cells to pair up.

In what follows, we set the ratio of the dissociation constant to the secretion rate (*K*_d_/*s*) at the value which gives the quickest mate-finding behaviour for D and SD cells (electronic supplementary material, figure S4). This ratio is critical because it determines whether a chemotactic response by detecting cells (dictated by *K*_d_) to the chemical profile generated by signalling cells (dictated by *s*; equations (2.2) and (2.3)) is possible. Consistent with experimental reports [35,36], cells cannot detect signals below a range of values for this ratio, and signal molecules saturate membrane receptors above a range (electronic supplementary material, figures S2 and S4).

### 3.1. Variation in the movement persistence

**Figure 3.**
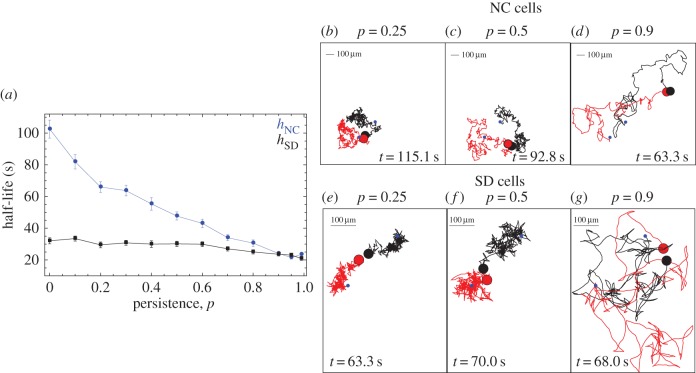
High persistence minimizes half-life of non-chemotactic and secrete-and-detect cells. (*a*) Mean half-life (averaged over 40 simulations) against persistence for non-chemotactic cells (blue) and secrete-and-detect cells (black). Example trajectories of two cells until they meet for (*b–d*) non-chemotactic cells and (*e–g*) secrete-and-detect cells. Initial positions are spaced equally far apart (blue dots). The persistence parameter for each simulation is indicated at the top of each plot. The duration of the search (*t*) is given at the bottom of each square. Other parameters: (*ρ*_0_, *s*, *s/K*_d_, *u*, *d*, *α, v*) = (5.1 × 10^6^ cells m^−2^, 1 s^−1^, 10^−4^, 10^−3^ s^−1^, 40 µm, 100, 100 µm s^−1^).

Variation in the persistence parameter, *p*, indicates how likely cells are to maintain their directionality at each time step in the simulation. We plotted the half-life for NC cells against *p* (figure 3*a*). Cells pair more quickly as their persistence increases. This is because larger *p* results in an increase in the space investigated by cells within a fixed time period, which increases their chance to meet one another (figure 3*b–d*).

The behaviour of SD cells differs qualitatively from that of NC cells (figure 3*a*). SD cells secrete and respond to a chemoattractant and so migrate towards one another. However, directional movement is inhibited as SD cells move around their local trail during migration (figure 3*e–g*), as anticipated (figure 1), echoing the findings of Taktikos *et al.* [31]. It follows that SD cells experience a trade-off between movement inhibition and directional migration as *p* increases (figure 3*a*,*e,f*). This gives SD cells a large advantage over NC cells at low values of *p.* For higher values of *p,* SD cells less frequently reorient their movement according to the chemical gradient. This reduces but does not eliminate the negative effect of self-inhibition, yet their capacity for directed migration remains compromised (figure 3*e* cf. 3*f,g*). Therefore, SD cells cannot exploit variation in *p* to optimize their search. This contrasts with NC cells that benefit greatly from higher persistence and even do slightly better than SD cells at extreme value of persistence, when *p* > 0.9 (figure 3*a*).

**Figure 4.**
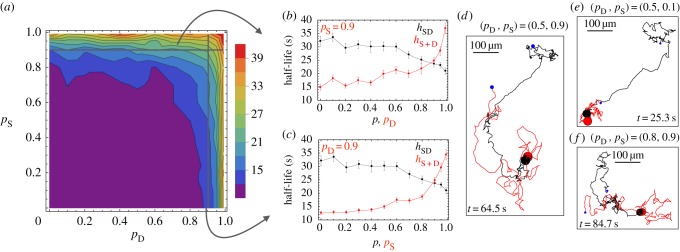
Low persistence minimizes half-life for separate secrete-only and detect-only cells. (*a*) Heat map of the mean half-life (averaged over 40 simulations) for secrete-only and detect-only cells (*h*_S*+*D_) given different values of persistence for secretors (*p*_S_) and detectors (*p*_D_). (*b*) The half-life (mean ± s.d. of 40 simulations) for secrete-only and detect-only cells (red) is compared with the half-life of secrete-and-detect cells for variable persistence *p* (black) for fixed secretor (*p*_S_ = 0.9) and variable detector persistence, and (*c*) for fixed detector (*p*_D_ = 0.9) and variable secretor persistence. (*d–f*) Example trajectories of two cells, one secretor and one detector, given different *p*_S_ and *p*_D_ values. Initial positions are spaced equally far apart (blue dots), with the duration of the search (*t*) given at the bottom of each square. Other parameters: (*ρ*_0_, *s*, *u*, *d*, *α, v*) = (5.1 × 10^6^ cells m^−2^, 1 s^−1^, 10^−3^ s^−1^, 40 µm, 100, 100 µm s^−1^). The ratio *s/K*_d_ is set equal to 10^−4^ and 10^−5^ for SD and S + D cells, respectively.

If the population consists of detect-only (D) and secrete-only (S) cells, with independent persistence parameters *p*_S_ and *p*_D_, respectively, we observe exactly the opposite effect of persistence (figure 4*a–c*). Smaller values of *p*_D_ and *p*_S_ are beneficial. At lower values of *p*_D_, D cells reorient themselves according to the chemoattractant more frequently. This results in swifter migration towards secreting cells and shorter search times (figure 4*d,f*). S cells with lower *p*_S_ stay in a local area. This has two advantages—it generates a stronger signal towards which D cells can orient, and increases the correlation between the signal and the position of the signaller, making the S cell a clearer target for detecting cells (figure 4*d,e*). In both cases, as persistence increases, cells change their orientation less frequently and this is disadvantageous as they either fail to generate a strong and predictive local signal to which others are attracted (S cells) or over-shoot the source of a signal (D cells). Importantly, the optimal half-life *h*_S+D_ lies below that for both *h*_SD_ and *h*_NC_, suggesting that secrete-only plus detect-only cells can optimize their search beyond the optimal searches of both non-chemotactic and secrete-and-detect cells. This holds true even when homogamous pairings between secrete-only and detect-only cells are forbidden; a stringent condition as this means half the cell pairings in the population are forbidden. For large *p* the effect of chemotaxis is diminished as cells do not update their direction chemotactically frequently enough, and the value of *h*_S*+*D_ rises above *h*_NC_ and *h*_NC_ (figure 4*b,c*). This disadvantage arises from the restriction that secrete-only plus detect-only cells cannot mate with each another.

### 3.2. Cell speed and the diffusion constant

Variation in the cell speed, *v*, naturally affects the half-life values. The faster cells move, the faster they form pairs, resulting in lower half-life values independent of chemotaxis. So we assess the relative efficiency of the two modes of chemotactic movement by comparing the ratios *h*_SD_/*h*_NC_ and *h*_S*+*D_/*h*_NC_, with ratios below one indicating that chemoattraction is favourable.

**Figure 5.**
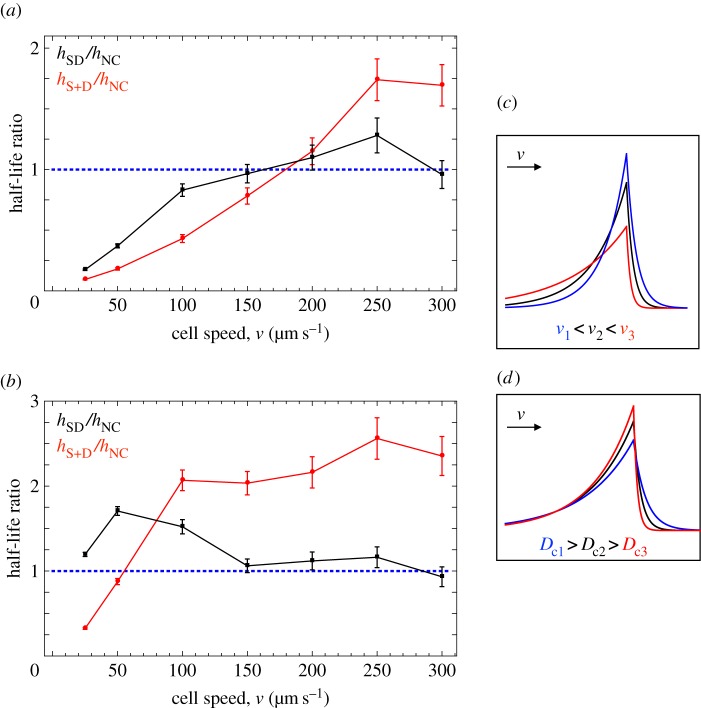
Diffusion and speed. Mean half-life ratios with error bars (averaged over 40 simulations) for secrete-only and detect-only cells (red) compared with non-chemotactic (blue dotted) and secrete-and-detect (black) cells, for varying cell speed given (*a*) high diffusion *D*_c_ = 10^−5^ cm^2^ s^−1^ and (*b*) low diffusion *D*_c_ = 10^−6^ cm^2^ s^−1^. Values below the blue line indicate that chemotaxis confers an improvement in the rate of pair formation. The asymmetry of chemical concentration around a moving secretor (direction indicated by the arrow) is greater as the secretor's speed (*v*) increases and the chemical diffusivity (*D*_c_) decreases (illustrated in a single dimension in (*c*) and (*d*), respectively). Other parameters (*ρ*_0_, *s*, *u*, *d*, *α*) = (5.1 × 10^6^ cells m^−2^, 1 s^−1^, 10^−3^ s^−1^, 40 µm, 100). The ratio *s/K*_d_ is set equal to 10^−4^ and 10^−5^ for SD and S + D cells, respectively. The persistence parameter for NC and SD cells is set equal to 0.2. For S + D cells, we set (*p*_S_, *p*_D_) = (0.2, 0.5).

When the diffusion coefficient equals 10^−5^ cm^2^ s^−1^ and *v* < 200 µm s^−1^, both SD and S + D cells find partners quicker than NC cells (*h*_SD_/*h*_NC_ and *h*_S*+*D_/*h*_NC_ > 1; figure 5*a*). The advantage of chemotaxis declines as cell speed increases and the two ratios exceed 1 once *v* = 200 µm s^−1^ (figure 5*a*). This is because of a number of factors. When cells move fast they meet each other more frequently, purely by chance. This benefits NC cells more as they rely on random collisions to find partners. SD and S + D cells, on the other hand, rely mainly on chemotaxis. Faster movement weakens the correlation between the chemical signal and the position of the secreting cell, thereby reducing the effect of chemoattraction in bringing cells together for SD and S + D cells. So there is a subtle interplay between the efficiency of chemotaxis and cell speed. S + D cells do better than SD cells for *v* < 200 µm s^−1^ (*h*_S*+*D_/*h*_NC_ < *h*_SD_/*h*_NC_; figure 5*a*). This is because of the interference of SD cell receptors with the cell's own signalling molecules which is amplified with cell speed (figure 5*c*). So at high speed, the advantage of chemoattraction disappears (*h*_SD_/*h*_NC_ = 1 when *v* = 200 µm s^−1^). With the relative benefits of chemotaxis becoming weaker as speed increases, the restriction of S and D cells to nonhomogeneous pairings becomes significant and S + D cells do worse than SD cells for *v* > 200 µm s^−1^.

These effects are amplified for smaller diffusion coefficients (figure 5*b*, *D*_c_ = 10^−6^ cm^2^ s^−1^). S + D cells now perform well only at slow speeds *v* ≤ 50 µm s^−1^, and SD cells appear to have no advantage at all over NC cells (figure 5*b*). Importantly, the chemotactic prowess of cells relies on the diffusion coefficient. *D*_c_ specifies the speed at which the chemoattractant diffuses away from a secreting cell. It follows that the correlation between the signal and the secreting cell's position becomes weaker for lower *D*_c_ (for fixed cell speed), impairing the search of both SD and D cells. Moreover, the tail of high concentration behind a moving secretor becomes more pronounced for small values of *D*_c_, explaining why SD cells perform so poorly (figure 5*d*). SD cells behave like NC cells once *v* > 150, and chemotaxis is effectively redundant.

The distinction between *h*_SD_/*h*_NC_ and *h*_S*+*D_/*h*_NC_ even for low cell speed and high *D*_c_ (10^−5^ cm^2^ s^−1^) indicates that saturation of receptors in SD cells also holds a key role in restricting the performance of SD cells. Receptors on SD cells will inevitably be occupied to some extent by their own pheromone, weakening the signal perceived from a remote signaller (electronic supplementary material, figure S3). A quantitative account for this phenomenon is provided in the electronic supplementary material.

### 3.3. Chemotactic constant

In the analysis above, we fixed the chemotactic constant at *α* = 100. This parameter determines how the external information the cell receives (chemical gradient, number of occupied receptors) is turned into a cellular response (change in direction of movement). Cells with a higher value of *α* are more responsive to the environmental gradient in the chemical signal (see Methods and model outline)*.* Eukaryotic cells can amplify very weak external signals, suggesting that *α* can be very large [51,52].

**Figure 6.**
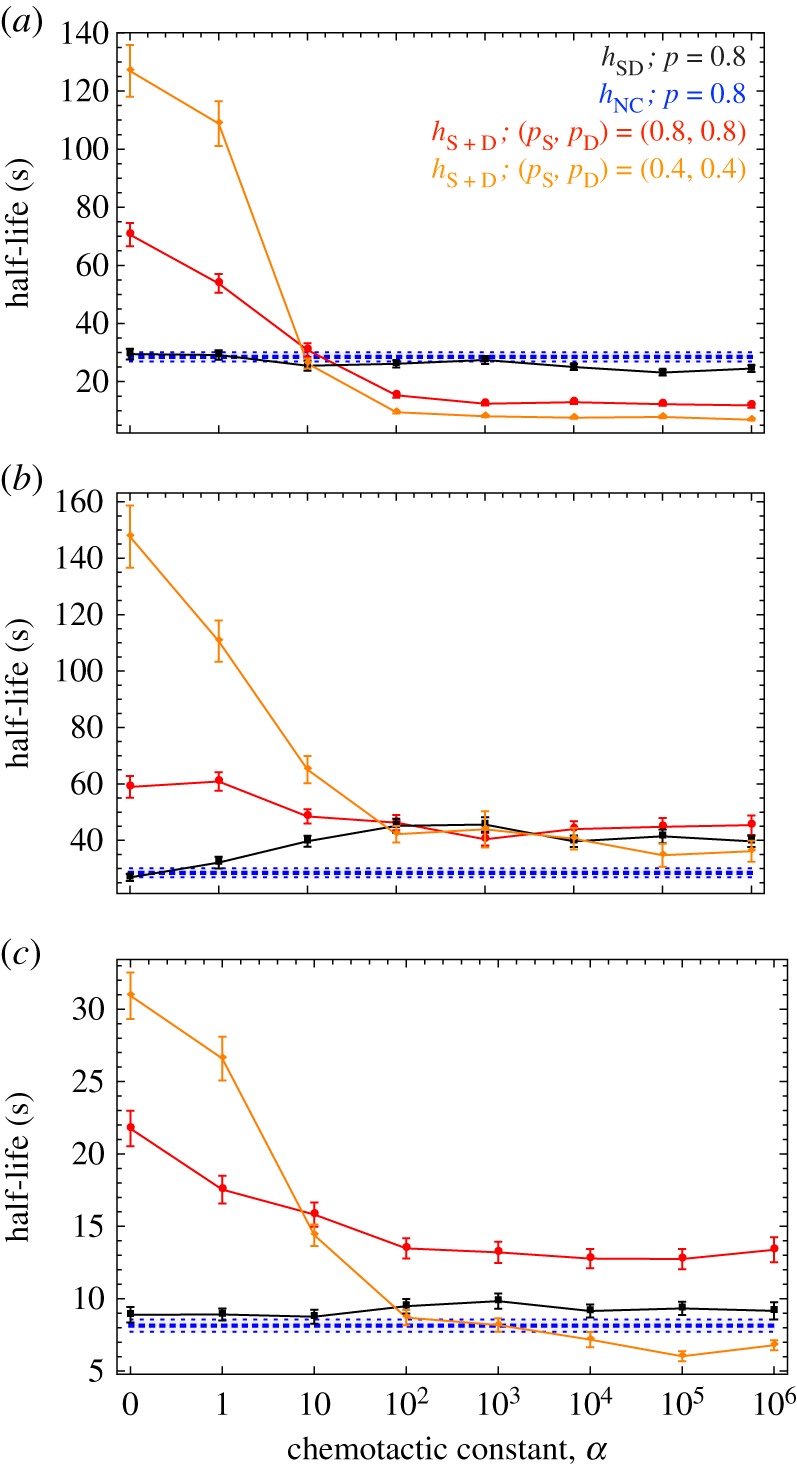
The half-life against the chemotactic constant. The half-life for secrete-only and detect-only cells (red and orange) compared with non-chemotactic (blue dotted), and secrete-and-detect (black) cells (mean ± s.d. of 40 simulations), for variation in the chemotactic constant (*α*) under (*a*) intermediate cell speed *v* = 100 µm s^−1^ and high diffusion coefficient *D*_c_ = 10^−5^ cm^2^ s^−1^, (*b*) intermediate cell speed *v* = 100 µm s^−1^ and low diffusion coefficient *D*_c_ = 10^−6^ cm^2^ s^−1^ and (*c*) high cell speed *v* = 250 µm s^−1^ and high diffusion coefficient *D*_c_ = 10^−5^ cm^2^ s^−1^. Non-chemotactic cells are not affected by the value of *α*. Values below the blue line indicate that chemotaxis confers an improvement in the rate of pair formation. Other parameters: (*ρ*_0_, *s*, *u*, *d*) = (5.1 × 10^6^ cells m^−2^, 1 s^−1^, 10^−3^ s^−1^, 40 µm). The ratio *s/K*_d_ is set equal to 10^−4^ and 10^−5^ for SD and S + D cells, respectively.

Here, we consider how different values of *α* impact on our findings. We begin by examining a case of intermediate speed and diffusivity. The half-life for SD cells is equal to that for NC cells when *α* = 0, and decreases slightly as *α* increases (figure 6*a*). By contrast, S + D cells have a much longer half-life for small values of *α* because the separation of secretion and detection reduces the density of fusible cells (as mentioned above). But the S + D half-life decreases sharply as *α* increases, dropping below *h*_SD_ and *h*_NC_ for *α* ≥ 100 (figure 6*a*). It follows that D cells, and so S + D pairings, benefit greatly by increasing their chemotactic responsiveness. This contrasts with SD cells, because as they become more sensitive to the overall gradient they also become more sensitive to the concentration they themselves produce.

This advantage of chemotaxis is less evident at lower diffusivity (figure 6*b*). For low diffusion of the signal, increasing the chemotactic constant has a negative effect on SD cells. This is because of the greater movement inhibition on SD cells which is amplified as *α* increases (figure 6*b*). Conversely, the search capability of D cells improves with higher *α* even for low *D*_c_. Further improvement can be achieved by modulation of *p*_S_ and *p*_D_ along with *α* (figure 6*b*). However, even for this optimal parametrization NC cells outperform S + D cells. This brings into question the effectiveness of chemoattraction when fast-moving cells employ signals that diffuse very slowly (also see figure 5*b*). A similar picture appears with higher speed (figure 6*c*). SD cells perform only slightly worse than NC cells but *α* has little effect on their search. S + D cells, on the other hand, can achieve an optimal half-life which outperforms NC cells for high *α* but only with appropriate *p*_S_ and *p*_D_ values. These observations indicate that SD cells perform poorly, but also that they have limited capacity to alter their chemotactic response and so improve their performance. On the other hand, in an S + D system, both S and D cells can increase their sensitivity to the chemical field, or vary their persistence and still improve performance.

### 3.4. Cell size

**Figure 7.**
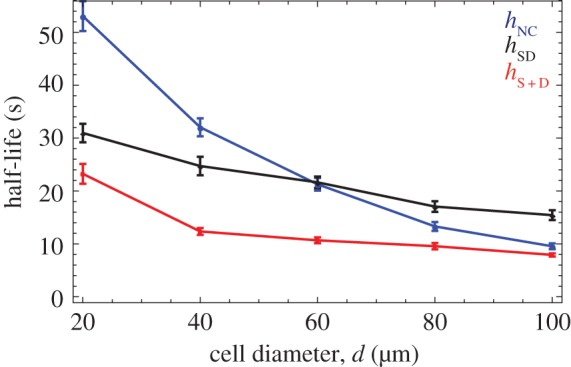
The effect of cell size. The half-life of non-chemotactic (blue line), secrete-and-detect cells (black line) and for secrete-only and detect-only cells (red line) against cell diameter (mean ± s.d. of 40 simulations). Other parameters: (*ρ*_0_, *s*, *u*, *d*, *α*) = (5.1 × 10^6^ cells m^−2^, 1 s^−1^, 10^−3^ s^−1^, 40 µm, 100). The ratio *s/K*_d_ is set equal to 10^−4^ and 10^−5^ for SD and S + D cells, respectively. The persistence parameter for NC and SD cells is set equal to 0.2. For S + D cells, we set (*p*_S_, *p*_D_) = (0.2, 0.5).

In the previous sections, cell diameter, *d*, was fixed at 40 µm. We varied cell diameter from 20 µm (indicative of small protists such as yeasts) up to 100 µm (indicative of large algae or ciliates). As cell size increases, the half-life for NC cells declines and does so more rapidly than for SD and S + D cells (figure 7). NC cells make no use of chemical signals, and rely on random collisions to find mating partners. They thus benefit from increases in cell diameter. SD and S + D cells rely mainly on chemotaxis to find partners and less on random collisions and so gain less from increases in cell diameter. Furthermore, SD cells benefit less from increases in cell size compared with S + D cells, and *h*_SD_ > *h*_NC_ for *d* > 60 µm. This suggests that inhibition due to self-secretion in SD cells probably increases with cell size.

## 4. Discussion

Signalling interactions between gametes are fundamental for sex. They entail both diffusible and surface bound signals that serve for partner attraction and recognition, and gamete communication during fusion [26,53–55]. These interactions are nearly always asymmetric so that gametes send and receive signals in a mating-type- or sex-specific manner. In this work, we ask whether asymmetric signalling enhances the efficiency of the signalling interaction itself, a theory first proposed in the 1980s [23].

Some general principles emerge from our analysis. Non-chemotactic cells can improve their search for a partner when they move in fixed directions for longer periods of time (high persistence, figure 3*a*). That straight-line movement can optimize a random search has been shown before in a different context (see Li *et al.* and references therein [33]). When cells are unable to maintain a fixed directionality for prolonged periods, symmetric chemotaxis (all cells send and receive the same signal) can improve pairing rates compared with non-chemotactic cells (figure 3*a*). This benefit occurs under a limited range of conditions, in particular when cells are relatively small, move with low to intermediate speed and chemoattractant diffusion is fast (figure 5*a*). The limited capacity of secrete-and-detect cells to optimize their search arises from self-inhibition. For fast-moving cells, this is mainly because of a self-induced asymmetry in chemical concentration that accumulates behind secreting cells, causing them to reorient away from the direction of the external signal. Even for immotile species (such as yeasts) or cells moving at very low speed (such as amoebae [56]), we predict that the performance of secrete-and-detect cells will be compromised, mainly because of saturation of their receptors by their own pheromone.

By contrast, substantial improvement in partner attraction and pair formation occurs when cells have asymmetric roles in sexual chemotaxis, across a much wider range of parameter values. When gametes either secrete or are attracted to a pheromone but not both, they avoid the loss in performance because of self-inhibition. Both detectors and secretors are able to exploit variation in their capacity to attract or detect other cells respectively. Detectors can find secreting cells faster when they frequently update their orientation according to the chemical gradient and when their chemotactic sensitivity is high. Eukaryotic cells are able to amplify very weak external signals (shallow gradients) [51,52], suggesting that very large values for the chemotactic constant are possible. The attracting capacity of secretors improves when they move slowly or reduce their persistence, which both increase the association between their position and signal. Improved mating rates with gametic differentiation follow even when cells with the same roles (detectors and secretors) are associated with distinct mating types that preclude pairing, and so halve the number of potential mating partners. These results suggest that asymmetric gamete roles during sexual chemotaxis can be crucial, even in the absence of morphological differences and anisogamy.

Our findings are important because they explicitly quantify the efficiency of sexual chemotaxis under a range of parameter values that allow us to interpret their relevance to a range of protists and their gametes (table 2). Cell speed, the chemoattractant diffusion coefficient and cell size dictate the impediment conferred with symmetric signalling, and the extent to which sexual chemotaxis can be beneficial. At low diffusion coefficients only slow moving cells can efficiently use chemotaxis implying that signals secreted by faster moving cells, such as some algae and ciliates, should be associated with higher diffusion coefficients. Slower cells, on the other hand, such as amoebas, yeasts and diatoms can afford to use signals that are less diffusible and indeed do so (table 2). If cell encounters are frequent without chemotaxis, as is the case when cell speed exceeds a threshold, the necessity for chemotactic partner attraction becomes ambivalent. *Paramecia* and *Tetrahymena*, for example, can reach very high speeds and effectively form pairs without the use of chemotaxis [57], although other ecological parameters such as the cell density also play a role (see cell density section and sensitivity analysis in the electronic supplementary material). Finally, we expect simultaneous secretion and detection to be more problematic for larger cells (such as amoebas and ciliates).

Many protists including examples from fungi [26], algae [27,58] and ciliates [28] have mating-type-specific pheromones and receptors. Frequently, both mating types produce a pheromone/receptor pair with receptors that are only sensitive to the pheromone of the opposite type. In our model, we only considered a single pheromone/receptor pair, but the same principles are likely to apply to bi-directional signalling, as long as pheromones and receptors within the same cell are incompatible. In fact, we anticipate pair formation to be faster if each mating type has their own pheromones and receptors for the opposite type since attraction would be mutual as opposed to one-sided. A further complexity is that some species retain multiple mating types (not just two). Even then, each type synthesizes its own pheromone and receptors that are responsive to all or some non-self pheromones but never its own. This has been well documented in some ciliates and fungi [28,59]. What determines the number of mating types and the specificity of their receptors needs to be investigated in the context of signalling examined here.

Our model does not consider evolutionary transitions, for example, from secrete-and-detect to secrete-only or detect-only cells, or the origins of secretion and detection. However, our work is important to understand the underpinnings of sexual signalling and can form the basis for an evolutionary analysis. This is not a trivial question to address in the biophysical framework developed here, but our findings point to some clear constraints. For instance, movement in secrete-and-detect cells is inhibited, suggesting that they will have difficulties finding partners in a wild-type non-chemotactic population when introduced at low frequency. This indicates that such cells were unlikely progenitors in the evolution of sexual chemotaxis. It also questions the significance of studying a transition from a secrete-and-detect to a secrete-or-detect population, as modelled by Hoekstra [23]. Another finding implicit in our modelling is strong frequency-dependence between secretor and detector, as the more secretor cells in a population the greater is the advantage of detectors and vice versa. Therefore, we predict the ‘fitness' of secrete-only and detect-only cells will increase as the relative frequency of the opposite type increases. Also, we note that eukaryotic cells produce and respond to diffusible molecules for reasons other than sexual chemotaxis, such as aggregation [60] and finding food [61]. So it is worth thinking how secrete-only or detect-only mutants could activate and/or modify pre-existing pathways as opposed to inducing de novo synthesis.

Finally, it is important to also consider the significance of asymmetric signalling in organisms with mating types that show no evidence of sexual chemotaxis such as *Paramecium* [57]. Many protists, for example, are thought to use diffusible signals to instigate differentiation into sexually competent cells [27] or to coordinate conjugation [54]. Recent experiments have found that between-cell communication through diffusible cues becomes challenging when cells secrete and sense the same chemical [62]. Along the lines of our work, this is because remote signals are undermined when contrasted to self-signalling. Gametes also use membrane bound signals for recognition, adhesion and fusion [63]. We did not address the significance of an asymmetry in membrane bound interactions, which requires substantially more attention. Possible issues could arise when ligands and receptors on the same cell bind to one another, instigating unwanted processes in the absence of a partner, or saturating receptors. The effects of this would depend on the dynamic geometry of the cell, the diffusion of ligands and receptors on the cell membrane, trade-offs between binding specificity and promptness of the interaction, and possible mechanisms via which cells could avoid self-binding. We plan to address these questions theoretically and experimentally in future work.

## Supplementary Material

Derivation of main equations, further methods and stability analysis
