# Supplementary material for: Cell–cell signalling in sexual chemotaxis: a basis for gametic differentiation, mating types and sexes
Source: J R Soc Interface. 2015 Aug 6;12(109):20150342. doi: 10.1098/rsif.2015.0342 (PMC4535405; doi:10.1098/rsif.2015.0342)
Supplement: Derivation of main equations, further methods and stability analysis [file rsif20150342supp1.pdf]

SUPPORTING INFORMATION

Cell-Cell Signalling in Sexual Chemotaxis: a Basis for Gametic Differentiation and the Evolution of Mating Types and Sexes

Zena Hadjivasiliou, Yoh Iwasa and Andrew Pomiankowski

1 Derivation and solution to Equation (1)

The classical diffusion equation reads,

$$\frac{\partial C(\mathbf{x},t)}{\partial t} = D_c \nabla^2 C(\mathbf{x},t). \tag{S1}$$

Adding a degradation term gives,

$$\frac{\partial C(\mathbf{x},t)}{\partial t} = D_c \nabla^2 C(\mathbf{x},t) - uC(\mathbf{x},t), \tag{S2}$$

where u is the chemical degradation rate. We can modify Eq. (S2) to incorporate a stationary source at $\mathbf{x}_0 = (x_0, y_0)$ as follows,

$$\frac{\partial C(\mathbf{x},t)}{\partial t} = D_c \nabla^2 C(\mathbf{x},t) - uC(\mathbf{x},t) + s\delta(\mathbf{x} - \mathbf{x}_0), \tag{S3}$$

where the source releases the pertinent chemical at rate s and $\delta(.)$ is the Dirac delta function. The chemical field in our model is generated by n motile cells, which are equivalent to multiple moving sources. The appropriate equation describing the chemical field is obtained by adding a source contribution for each cell along its trajectory,[1]

$$\frac{\partial C(\mathbf{x},t)}{\partial t} = D_c \nabla^2 C(\mathbf{x},t) - uC(\mathbf{x},t) + s\sum_{j=0}^{j=n} I_j \delta[\mathbf{x} - \mathbf{x}_j(t)], \tag{S4}$$

which is Eq. (1) in the main text. Here, $\mathbf{x}_j(t)$ is the trajectory of the jth cell over time and I_j is an indicator function equal to 1 if the jth cell secretes the pertinent chemical and 0 otherwise.To solve Eq.

(S4) first consider the solution to Eq. (S1) given by,

$$C(\mathbf{x},t) = \frac{1}{4D_c t} exp\left(-\frac{\mathbf{x}^2}{4D_c t}\right), \tag{S5}$$

where we assume that the chemical is released at time $t = 0$. Now assume that $\hat{C}$ satisfies Eq.(S1) and let $C = \hat{C}e^{-u(t-t_0)}$. Taking partial derivatives with respect to t and $\mathbf{x}$ we have,

$$\frac{\partial C(\mathbf{x},t)}{\partial t} = \frac{\partial \hat{C}(\mathbf{x},t)}{\partial t} e^{-u(t-t_0)} - u\hat{C}e^{-u(t-t_0)}$$

and

$$\nabla^2 C(\mathbf{x},t) = \nabla^2 \hat{C}(\mathbf{x},t)e^{-u(t-t_0)}.$$

Solving for $C(\mathbf{x},t)$ and substituting in Eq.(S1) we obtain

$$\frac{\partial C(\mathbf{x},t)}{\partial t} = D_c \nabla^2 C(\mathbf{x},t) - uC(\mathbf{x},t)$$

and so C satisfies Eq.(S2). It follows that the time dependent solution to Eq.S4 is the following convolution with respect to t and $\mathbf{x}$,

$$\begin{aligned} C(\mathbf{x},t) &= \textstyle\int_0^t \int_{-\infty}^{\infty} d\mathbf{y} d\tau \frac{1}{4D_c(t-\tau)} exp\left(-\frac{[\mathbf{x}-\mathbf{y}]^2}{4D_c(t-\tau)}\right) e^{-u(t-\tau)} s \sum_{j=0}^{j=n} I_j \delta[\mathbf{x}-\mathbf{x}_j(\tau)] \\ &= \int_0^t d\tau \frac{s}{4D_c(t-\tau)} e^{-u(t-\tau)} \sum_{j=0}^{j=n} I_j exp\left(-\frac{[\mathbf{x}-\mathbf{x}_j(\tau)]^2}{4D_c(t-\tau)}\right), \end{aligned} \tag{S6}$$

which the solution Eq.(1) in the main text. Eq.(3) in the main text is simply obtained by differentiating (S6) with respect to $\mathbf{x}$. We solve Eq. (2) and (3) in the main text using numerical intergration. All code was wirtten on C++.

2 Modelling movement

Non-chemotactic (NC) and secrete-only (S) cells move randomly. We model the update in their direction of movement as shown on Fig. S1a. Assume that the cell in question is at $(x_0,\ y_0)$. The angle θ determines the new direction the cell will move along, is independent of the cell's current direction of movement, and is drawn from a uniform distribution, Unif $[0, 2\pi]$. l is the length of the step the cell will take and is randomly chosen from a Unif $[0, 2v\mu]$ distribution where μ is the time step in our simulation. It follows that the cell's updated position will be equal to $(x_0 + l\ \cos\theta,\ y_0 + l\ \sin\theta)$. The average length

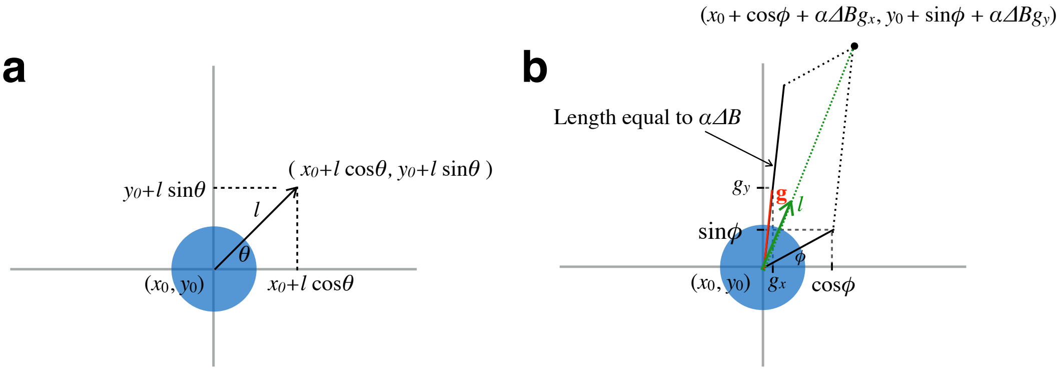

Figure S1: a: The position of a cell before, (x_0, y_0), and after taking a step in a random direction. The angle θ determines the random direction the cell will follow after reorientation and can take values in $[0, 2\pi]$. l is the length of the step taken by the cell and is ranomly chosen from a uniform [0, $2v\mu$] distribution. b: SD and D cells change their direction according to the chemical gradient. The vector **g** shown in red is a unit vector along the direction of the gradient. The cell updates it position by taking a step of length l along the direction of the dotted green vector which is the sum of a unit vector along a random direction and a magnified vector along the direction of the gradient. The greater this magnification (determined by $\alpha\Delta B$), the closer the direction the cell moves in is to the direction of the gradient. As before, l is chosen from a uniform distribution on [0, $2v\mu$].

of the step taken by a cell in time μ is equal to $v\mu$, and so the average speed per cell is equal to v (the cell speed in our model).

Detect-only (D) and secrete-and-detect (SD) cells have surface receptors and can become polarized along the chemical gradient **g**. We assume that polarization along the gradient depends linearly on the difference in receptor occupancy across the cells' polarized ends, ΔB, where,

$$\Delta B = \frac{C_{front}}{C_{front} + K_d} - \frac{C_{rear}}{C_{rear} + K_d}$$

and C_{front} and C_{rear} are the concentrations at the front and rear of the polarized cell respectively. D and SD cells change their position with probability 1-p_D and 1-p_{SD} so that the updated cell position will be a step of length l along the direction (x_0 + x_1 + $\alpha\Delta B g_x$, y_0 + y_1 + $\alpha\Delta B g_y$) where (x_0, y_0) is the position of the cell, (x_1, y_1) = (cosϕ, sinϕ) is a random unit vector with ϕ sampled from a Unif [0, 2π], and (g_x, g_y) is the unit vector in direction of the gradient found using Eq. (3). This is illustrated and explained in Fig. S1b.

At each time step we also add a noise term to the direction of movement for all cells, whether they change their direction randomly, chemotactically or just maintain their orientation. This is sampled from a $N(0, \pi/10)$ distribution and it is intended to incorporate extrinsic noise.

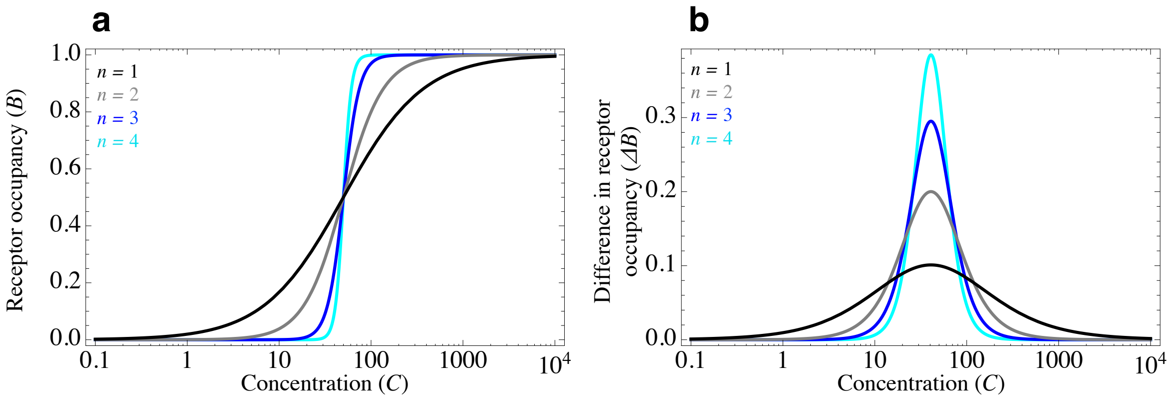

Figure S2: Receptor occupancy and difference in receptor occupancy across a polarized cell against concentration. a: Log scale plot of the Hill functions describing receptor occupancy against concentration for different values of the Hill coefficient n. b: Log scale plot of the difference in receptor occupancy across a cell's membrane against the average local concentration assuming an exponential concentration field (i.e. fixed $\Delta C/C$) for different values of the Hill coefficient n. The dissociation constant $K_d = 50$.

3 Receptor occupancy and saturation

Receptor occupancy can be quantified using Hill functions so that $B = \dfrac{C^n}{C^n + K_d}$ where B is the fraction of occupied receptors, C is the chemical concentration at the cell's surface, K_d is the constant of dissociation and n is the Hill coefficient that determines the interaction between the chemical and the receptor (Fig. S2). This formulation was first proposed by Hill[2] and has been subsequently applied broadly to pharmacological modelling,[3] and was used to model the ligand occupancy on eukaryotic cell membrane receptors during chemotaxis in experimental studies.[4] The difference in receptor occupancy across a polarized cell is given by,

$$\Delta B = \frac{C_{front}}{C_{front} + K_d} - \frac{C_{rear}}{C_{rear} + K_d} \tag{S7}$$

where C_{front} and C_{rear} are the concentration at the front and rear of the polarized cell respectively. ΔB is maximum for concentrations that maximize the gradient of B (Fig. S2). We assume a simple interaction between receptor and ligand, setting n=1.[4,5]

In the main text we show that moving secrete-and-detect cells (SD) suffer an impediment in their ability to search or be found by potential partners. This is for two reasons. Firstly, moving secretors end up with a tail of high concentration accumulating behind them, which urges them to move backwards while searching for potential partners and inhibits their migratory capacity. We quantified this impediment as a function of the key model parameters in the main text. A second issue is receptor saturation. We explicitly quantify the potential impact of receptor saturation by considering the net

signal perceived by a SD cell as opposed to a detect-only cell (D) (Fig. S3). Let ΔB_{SD} and ΔB_D be the difference in receptor occupancy across an SD and D cell respectively, and C^{SD}_{front}, C^{D}_{front}, C^{SD}_{rear}, C^{D}_{rear} be the concentration at the front and rear of the SD and D cell respectively. Then,

$$C^{SD}_{front} = C_0 + C_1, \tag{S8}$$

$$C^{SD}_{rear} = C_0 + C_2, \tag{S9}$$

$$C^{D}_{front} = C_1, \tag{S10}$$

$$C^{SD}_{rear} = C_2. \tag{S11}$$

Here C_0 is the concentration at the two ends of the SD cell due to its own pheromone (these are equal at the front and rear because the cell is not moving), C_1 and C_2 are the concentration due to the remote cell at the SD and D cells' front and rear respectively (Fig. S3a). Substituting (S8)-(S11) into (S7) and simplifying we obtain,

$$\Delta B_{SD} = \frac{K_d(C_1 - C_2)}{(C_0 + C_1 + K_d)(C_0 + C_2 + K_d)}, \tag{S12}$$

$$\Delta B_D = \frac{K_d(C_1 - C_2)}{(C_1 + K_d)(C_2 + K_d)}. \tag{S13}$$

Comparing (S12) and (S13), it is obvious that $\Delta B_{SD} < \Delta B_D$ (Fig.S3b). Differentiating (S12) and (S13) with respect to K_d, we find the optimal K_d (maximizing ΔB) to be equal to $\sqrt{C_0^2 + C_0C_1 + C_0C_2 + C_1C_2}$ and $\sqrt{C_1C_2}$ for SD and D cells respectively. Since $\sqrt{C_0^2 + C_0C_1 + C_0C_2 + C_1C_2} > \sqrt{C_1C_2}$, the optimal K_d should be larger for SD cells than for D cells (Fig.S3b). Our findings are in agreement with this prediction (Fig. S4 in following section).

4 Secretion rate and dissociation constant

In the main text we fixed the ratio of the dissociation constant (K_d) to the secretion rate (s). Here we consider variation in this ratio. The ratio K_d/s is critical because it determines whether the chemical profile generated by signalling cells (Eq. (2) in main text and Eq.(S6)) is detectable by the chemotactic capacity of detecting cells (K_d). We varied K_d/s for different baseline parameters and found that the same general picture emerges. A chemotactic response is possible for a range of intermediate values of K_d/s (Fig. S4). Below this, cells cannot detect the signal, and above this the signal molecule saturates

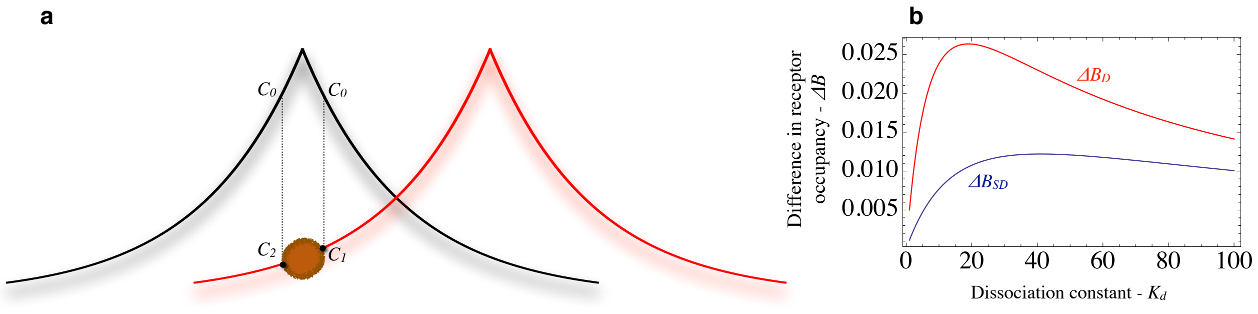

Figure S3: Illustration of receptor saturation. a: The cell shown in the diagram (brown circle) secretes a chemical whose concentration is indicated by the black exponential plot. The concentration due to its own secretion at the two polarized ends of the cell is equal to C_0. The red curve indicates the concentration due to a remote signaler so that C_1 and C_2 are the concentration of the remote signal at the front and rear of the polarized cell. b: The difference in receptor occupancy (ΔB) across a secrete-and-detect cell (blue) and a detect-only cell (red). ΔB_D is always higher than ΔB_{SD}.

the membrane receptors consistent with experimental reports.[4,5]

The qualitative behaviour of S+D cells is generally consistent across parameters when varying K_d/s. The only difference we observe is that the relative advantage of S+D against NC cells changes. This is largely as described in the main text and in the section that follows on cell density. S+D cells optimize their search for values of K_d/s around $10^{-5} - 10^{-4}$.

A range of different pictures emerges for SD cells. For parameters that are only mildly favourable to SD cells (e.g. high persistence, Fig. S4a, and large or low cell density, Fig. S4g, h) we only see a slight improvement, if at all, in the mating rates for SD cells across values of K_d/s. For favourable parameters (Fig. S4b, c, e) we see a drop in the half-life compared to NC cells that appears at $K_d/s \approx 10^{-4} - 10^{-3}$. Finally, for parameters that do not favour symmetric chemotaxis, such as high speed (Fig. S4d) and large cell size (Fig. S4f; also see section on cell size below), SD cells perform worse than NC cells when the value of K_d/s allows for a chemotactic response.

These observations are in agreement with the general patterns presented in the main text.

5 Boundary conditions and grid size

We simulate cell movement on a finite two-dimensional plane with periodic boundary conditions (Fig. S5). It follows that cells can move from one end of the grid to the other (space is continuous). Although the assumption of periodic boundaries may seem counter intuitive at first, it is equivalent to considering a window of cells at the center of a cell colony. Naturally, this would involve influences from cells that

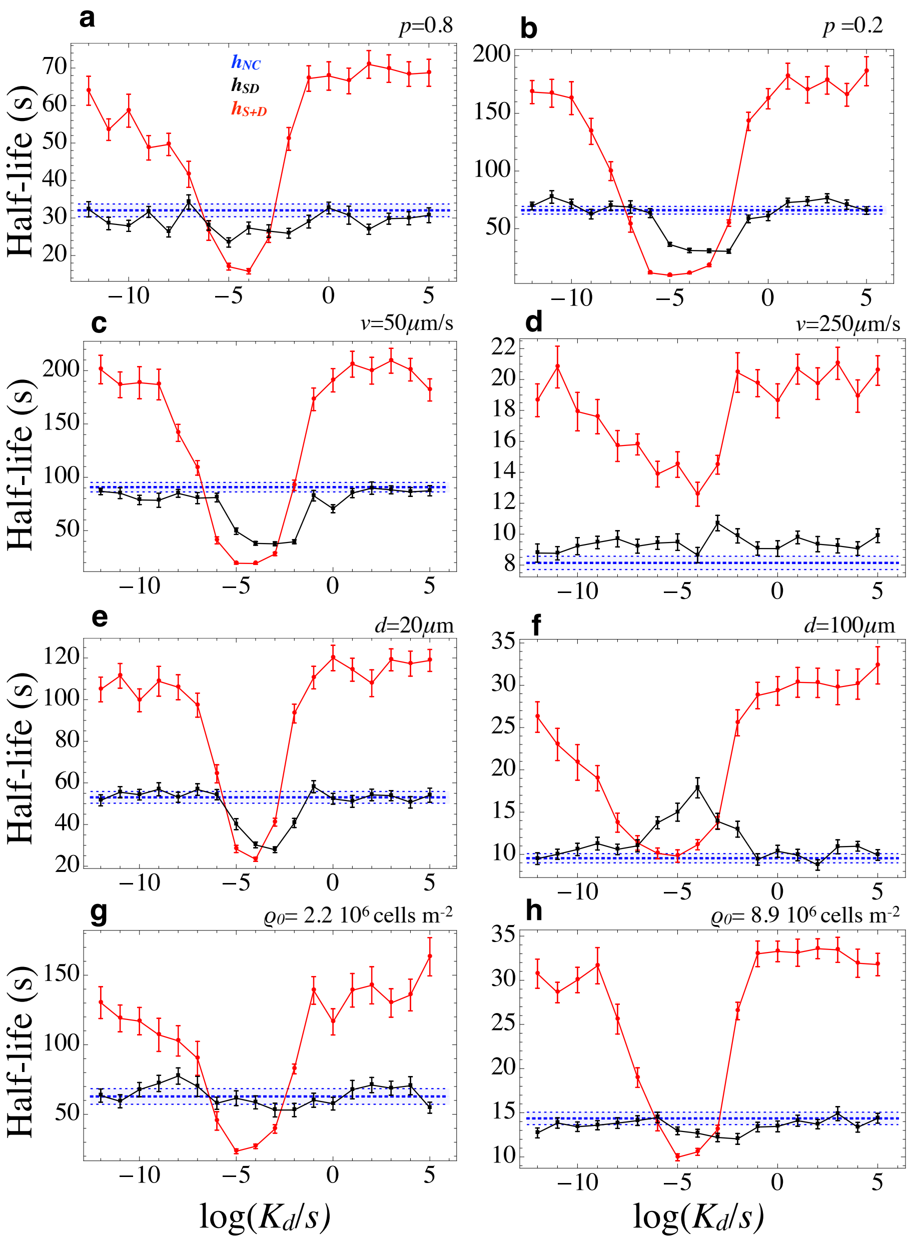

Figure S4: Half-life against the log ratio of the dissociation constant (K_d) to the secretion rate (s). The red and black lines are for h_{S+D} and h_{SD} respectively. The blue line and shaded regions indicate the mean and standard error for h_{NC}. Values below the blue region indicate more rapid pair formation.We repeated this for different parameter choices. As a baseline we used (ρ_0, s, u, d, α, p, p_S, p_D) = (5.1 10^6 cells m^{-2}, 1s^{-1}, 10^{-3}s^{-1}, 40μm, 100, 0.2, 0.2, 0.2). We repeated thew simulations 40 times.

are close to the edges of our window of interest, which are approximated by cells at the far opposite end (Fig. S5). We assume the same principle for molecular diffusion so that molecules of the diffusible pheromone can move from one side of the grid to the other and solve Eq. (2) and (3) from the main text using numerical integration. We implement this latter point by considering contributions from all 8 periodic reflections of each cell that contributes to Eq. (2) and (3). One important factor pertinent to periodic boundary conditions is the size of the plane considered. If its side is too small, it may fail to accurately emulate the environment of interest. In order to find an appropriate size for our grid we varied N (the side of the square plane) for high and low cell speed, high and low persistence and high and low chemical diffusivity and recorded the cell density decay over time for different cell speed and chemical diffusivity for NC cells, SD cells and S+D cells. Fig. S6 - Fig. S8 show the cell density decay with time starting from a fixed initial density but varying the grid size, cell size, persistence and cell speed. The lowest cell initial density we consider in our simulations is about 2 10^{-6} cells μm^{-2}. It follows from Fig. S6 - Fig. S8, that a grid with side equal to 3000μm is sufficient for the purposes of our study. All code was written on C++.

6 Variation in cell density and the chemical degradation rate

In the main text we varied movement persistence, cell speed, chemical diffusion coefficient, chemotactic sensitivity and cell diameter. The cell diameter, d, initial cell density, ρ_0, and chemical degradation rate, u, were kept fixed. Here we vary these parameters and show that their impact is merely quantitative.

Initial cell density, ρ_0, has an obvious effect on pairing rates – random cell encounters are more frequent the higher the initial density which leads to a less pronounced benefit for chemotaxis. We see that $h_{NC} > h_{SD} > h_{S+D}$ consistently across values of ρ_0 (Fig. S9). All three measures decrease exponentially as the initial cell density increases, albeit at different rates. The steepest drop occurs in h_{NC} and h_{SD}. So the ratio of h_{SD} to h_{NC} remains approximately fixed. This can be seen by comparing the slopes of the curves on Fig. S9. The slope of h_{S+D}, however, decreases more slowly than that of h_{NC} and h_{SD}, as the initial cell density rises. A simple explanation of these outcomes is that in an S+D population, cells cannot fuse with other cells of the same type. At high density, S and D cells meet other cells at random more frequently, and so reject half the potential partners. In contrast, SD and NC cells are free to pair up with any cells they encounter (this also depends on the cell speed, Fig. 4 in main text). The net effect is to reduce the advantage of separate S and D cells. At lower cell density,

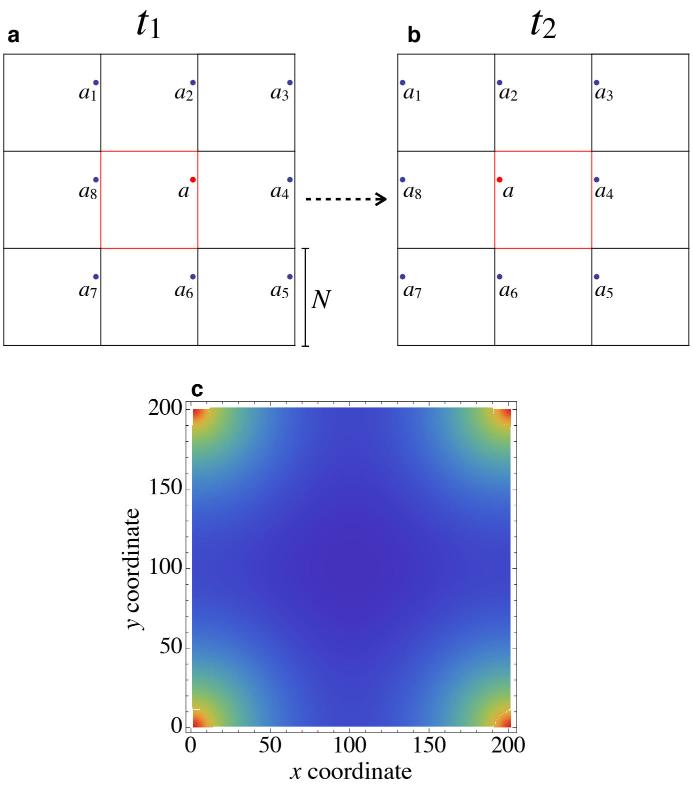

Figure S5: We implement periodic boundary conditions. a, b: The central red square is the main square where cells exist and move in our simulation. The eight surrounding squares are the periodic reflections of our main grid. The point a shown in red is repeated eight times at exactly the same position in the different squares. When a cell at a moves to the right from time t_1 to t_2, it exits the grid and re-enters it at the opposite end as illustrated in the diagram. c: The chemical concentration assuming N=200 and a single secreting cell at (0, 0). The assumption of periodic boundary conditions means that a cell secreting at (0, 0) will induce a high concentration at all four corners.

Non-chemotactic cells (NC)

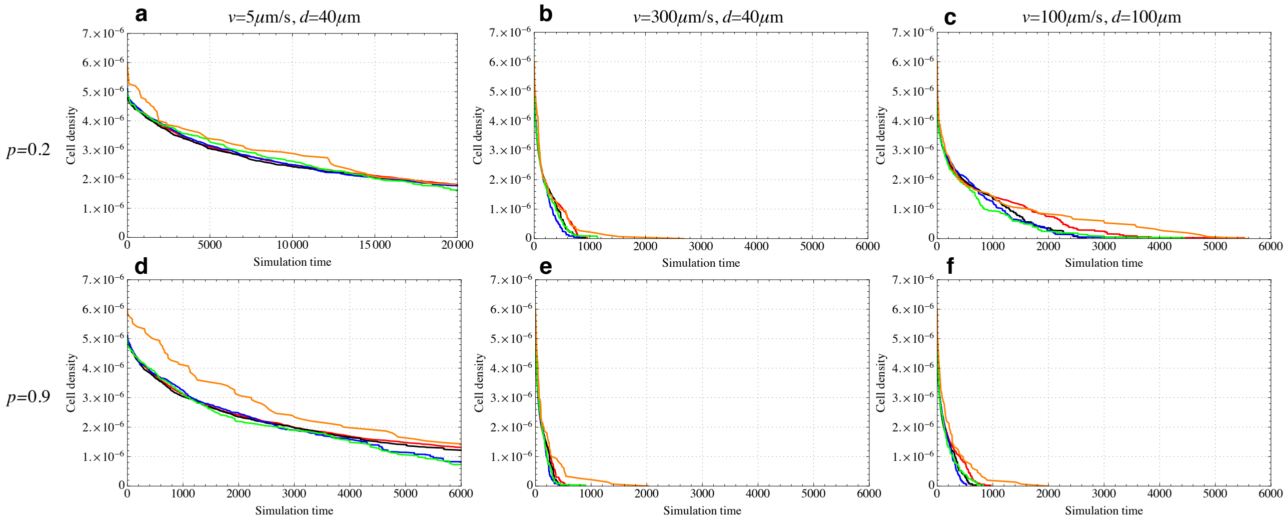

Figure S6: The cell density decay for varying grid size for nonchemotactic cells (NC). We repeated the simulation for varying cell speed, v, cell diameter. d and persistence, p, shown on each column and row. Color code: (Orange: N=1000, Green: N=2000, Blue: N=3000, Black: N=4000 Red: N=5000). Baseline parameters,$(s,\ u,\ \alpha) = (1\text{s}^{-1},\ 10^{-3}\text{s}^{-1},\ 100)$.Results were averaged over 30 simulations.

Secrete-and-detect cells (SD)

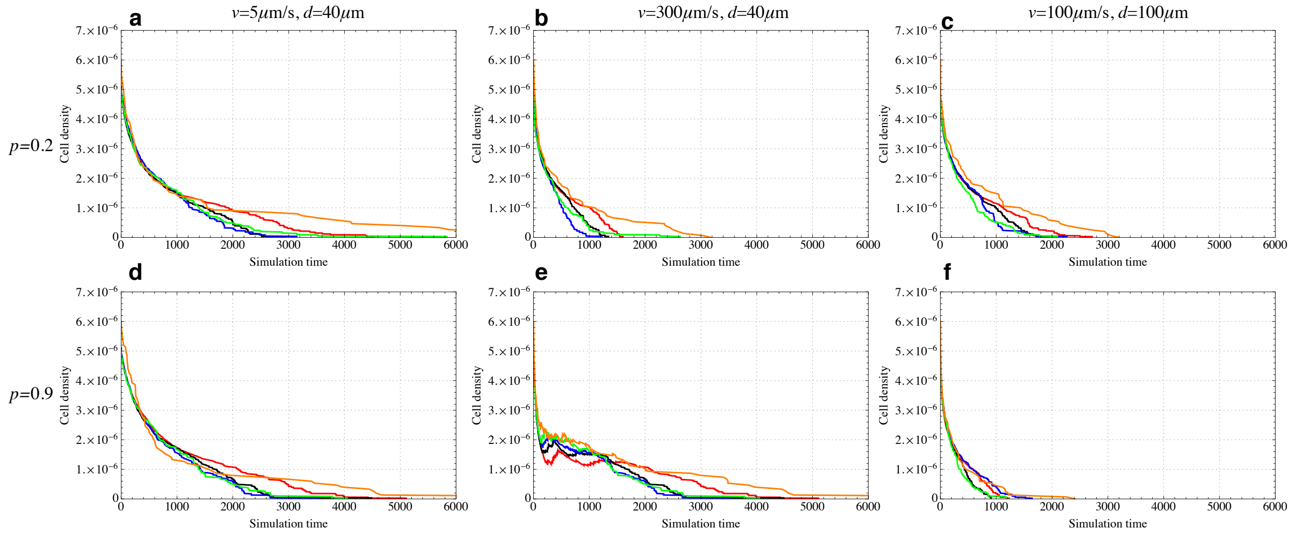

Figure S7: The cell density decay for varying grid size for secrete-and-detect cells (SD). We repeated the simulation for varying cell speed, v, cell diameter. d and persistence, p, shown on each column and row. Color code: (Orange: N=1000, Green: N=2000, Blue: N=3000, Black: N=4000 Red: N=5000). Baseline parameters,$(s,\ u,\ \alpha) = (1\text{s}^{-1},\ 10^{-3}\text{s}^{-1},\ 100)$.Results were averaged over 30 simulations.

Secrete-only and detect-only cells (S+D)

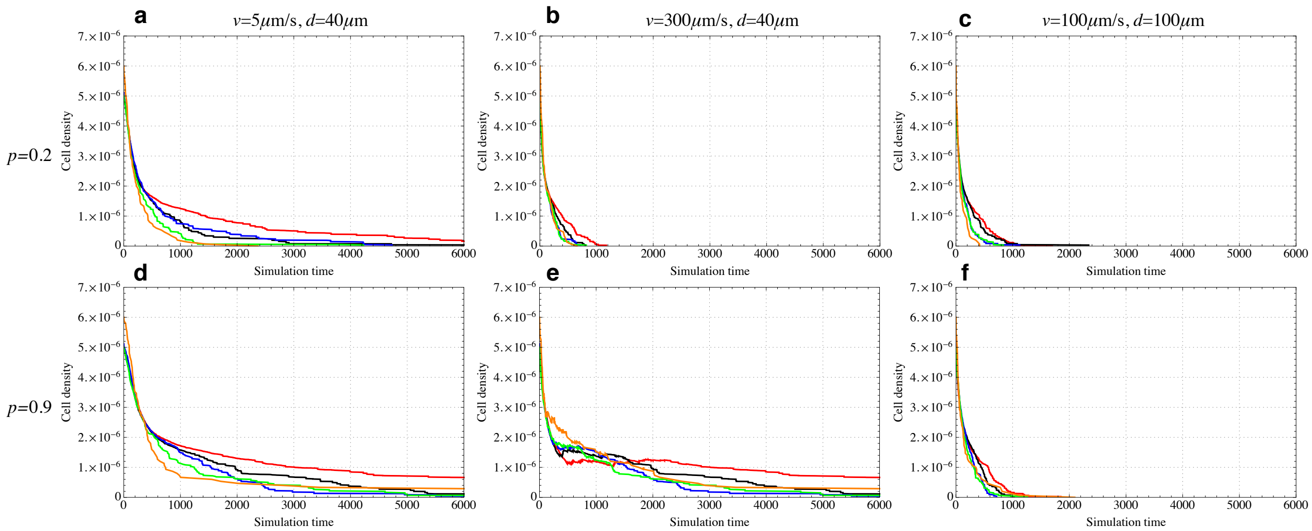

Figure S8: The cell density decay for varying grid size for secrete-only and detect-onnly cells (S+D). We repeated the simulation for varying cell speed, v, cell diameter. d and persistence, p, shown on each column and row. Color code: (Orange: N=1000, Green: N=2000, Blue: N=3000, Black: N=4000 Red: N=5000). Baseline parameters,(s, u, α) = (1s^{-1}, 10^{-3}s^{-1}, 100).Results were averaged over 30 simulations.

pheromone gradients have a larger effect on which cells meet, so the advantage of asymmetric signalling in a S+D population is very much higher. Cell density decreases within a single generation as gametes form pairs thereby reducing the number of gametes that are still free to mate. Therefore, even if the initial cell density is relatively high (both in terms of the number of cells and their size), the benefits of chemotaxis still are apparent but only after cell density falls.

We also varied the chemical degradation rate, u (Fig. S10). This parameter specifies the rate at which the diffusible pheromone in our model degrades in the environment. Fig. S10 indicates that the effect of this parameter is small as long as it is kept an order of magnitude below that of the secretion rate (the secretion rate, s, was taken to be equal to 1 for the purposes of Fig. S10). That is, as long as the pheromone is produced at a rate faster than the rate at which it is degraded, u has little impact on our results. When u exceeds s, no detectable signal can be generated and SD and S+D cells behave non chemotactically (Fig. S10). Note that when u=10, there is an increase in h_{SD}. This is because u is not large enough to eliminate the local concentration due to own secretion but significantly reduces remote signals and so SD cells suffer a larger disadvantage because of self-secretion. Also, recall that cells that homogeneous mattings are not allowed in an S+D world, explaining why h_{S+D} exceeds h_{NC} for high u.

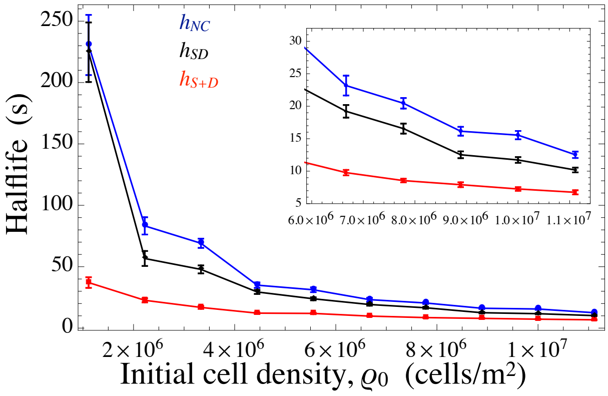

Figure S9: Half-life against initial cell density. The red, black and blue lines are for h_{S+D} , h_{SD} and h_{NC} respectively. Values were averaged over 40 simulations, the bars indicate the standard deviation. Baseline parameters: (ρ_0, s, u, d, α, p, p_S, p_D) = (5.1 10^6 cells m^{-2}, 1s^{-1}, 10^{-3}s^{-1}, 40μm, 100, 0.2, 0.2, 0.2).

7 Sensitivity analysis

We repeated the simulations that gave rise to Fig. 2, Fig. 3 and Fig.4 in the main text while varying the initial movement persistence, p, cell density, ρ_0, diameter, d, and speed, v.

The changes in Fig. S11 and Fig. S12, compared to Fig.2 and Fig.3 are mainly quantitative. Looking at Fig. S11, we see that h_{SD} is below h_{NC} across values of p when the cell size and speed are small (Fig. S11 a and c). Variation in the persistence does not result in significant changes in h_{SD} (as seen in Fig. 2), unless the cell size or speed are high. In these last instances (Fig. S11 b and d), low persistence is equivalent to frequent reorientation according to the gradient for SD cells. This is disadvantageous for larger and faster cells which optimize their search with high persistence, effectively adopting a behaviour equivalent to NC cells (explaining why h_{SD} decreases to become equal to h_{NC} in Fig. S11 b and d). These findings are in agreement with the results presented in the main text (e.g. see Fig. 4 and 6 for the effect of speed and cell size on SD cells). Variation in the persistence of secrete-only and detect-only cells using different baseline parameters does not change the qualitative behaviour seen in Fig. 3 in the main text (Fig. S12).

Similarly, the results presented in Fig. 4 in the main text are also robust to variation in the baseline parameters (Fig. S13). Any variation due to changes in p, ρ_0 and d are for reasons discussed in the main text and the preceding SI sections.

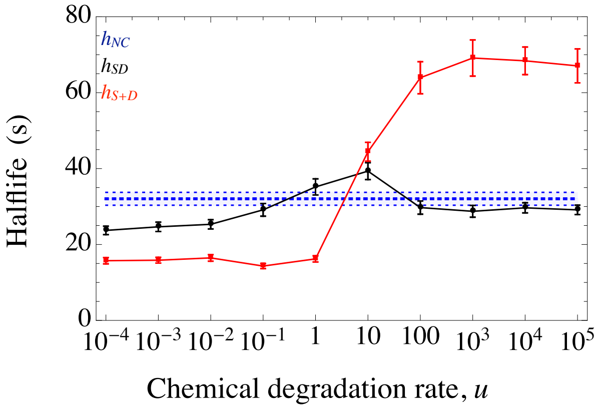

Figure S10: Half-life against the degradation rate, u. The red and black lines are for h_{S+D} and h_{SD} respectively. The blue line and shaded regions indicate the mean and standard error for hRW. Values were averaged over 40 simulations, the bars indicate the standard deviation. Baseline parameters: (ρ_0, s, u, d, α, p, p_S, p_D) = (5.1 10^6 cells m^{-2}, 1s^{-1}, 10^{-3}s^{-1}, 40μm, 100, 0.2, 0.2, 0.2). The ratio s/K_d is set equal to 10^{-4} and 10^{-5} for SD and S+D cells respectively.

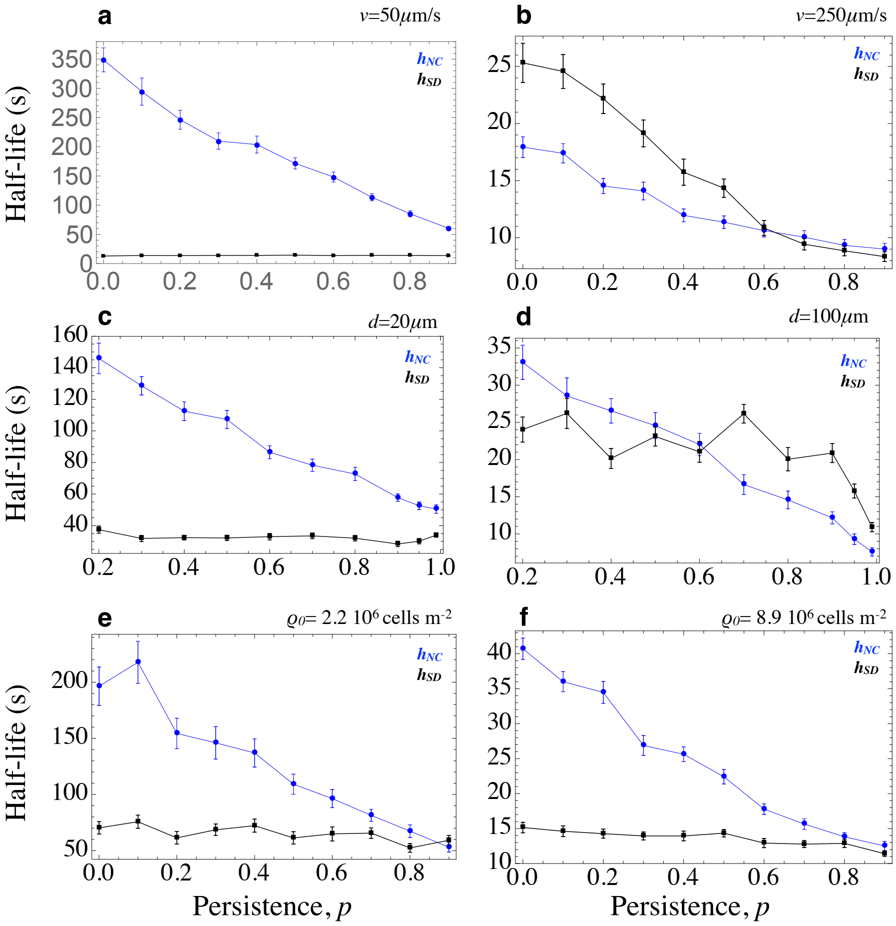

Figure S11: Mean half-life ratios with error bars (averaged over 40 simulations) for non-chemotactic cells (blue) and secrete-and-detect cells (black). a,b: for different cell speed, v, c, d: for different cell diameter, d and e,f: for different initial cell density ρ_0. As a baseline we used (ρ_0, s, u, d, α, p, p_S, p_D) = (5.1 10^6 cells m^{-2}, 1s^{-1}, 10^{-3}s^{-1}, 40μm, 100, 0.2, 0.2, 0.2). The ratio s/K_d is set equal to 10^{-4} and 10^{-5}.

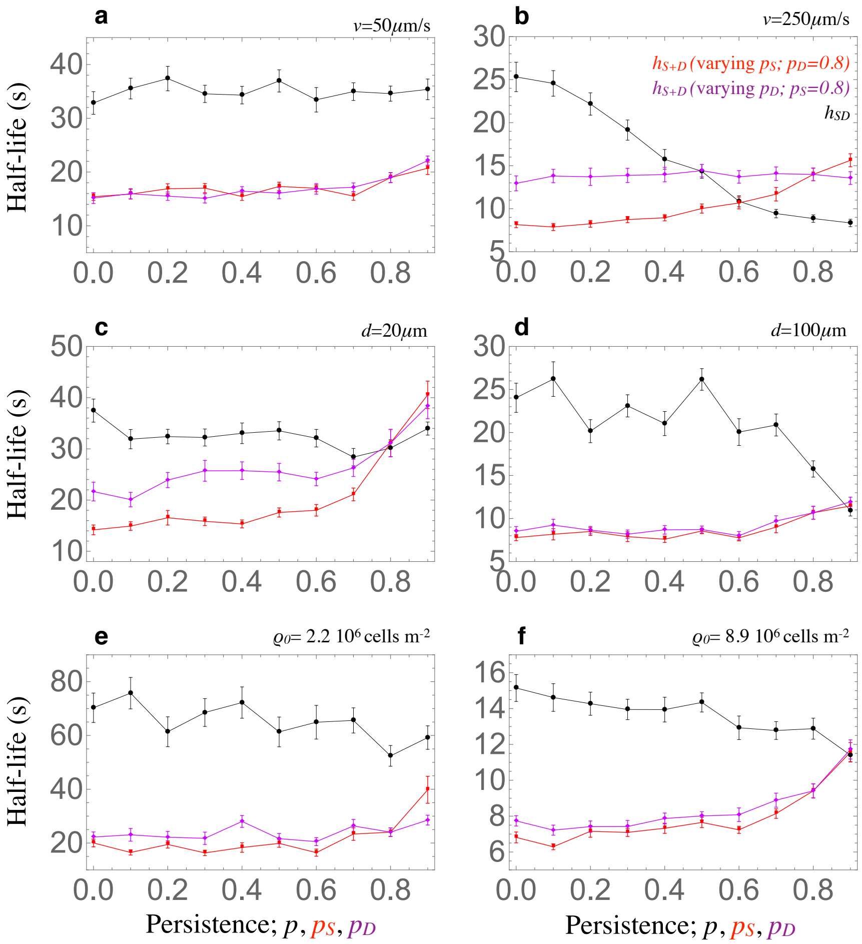

Figure S12: Mean half-life ratios with error bars (averaged over 40 simulations) for secrete-and-detect cells (black), and secrete-only and detect only cells varying p_S and keeping p_D at 0.8 (red), and varying p_D and keeping p_S at 0.8 (red). a,b: for different cell speed, v, c, d: for different cell diameter, d and e,f: for different initial cell density ρ_0. As a baseline we used $(\rho_0,\ s,\ u,\ d,\ \alpha,\ p,\ p_S,\ p_D) = (5.1\ 10^6$ cells m^{-2}, $1\mathrm{s}^{-1}$, $10^{-3}\mathrm{s}^{-1}$, 40μm, 100, 0.2, 0.2, 0.2). The ratio s/K_d is set equal to 10^{-4} and 10^{-5} for SD and S+D cells respectively.

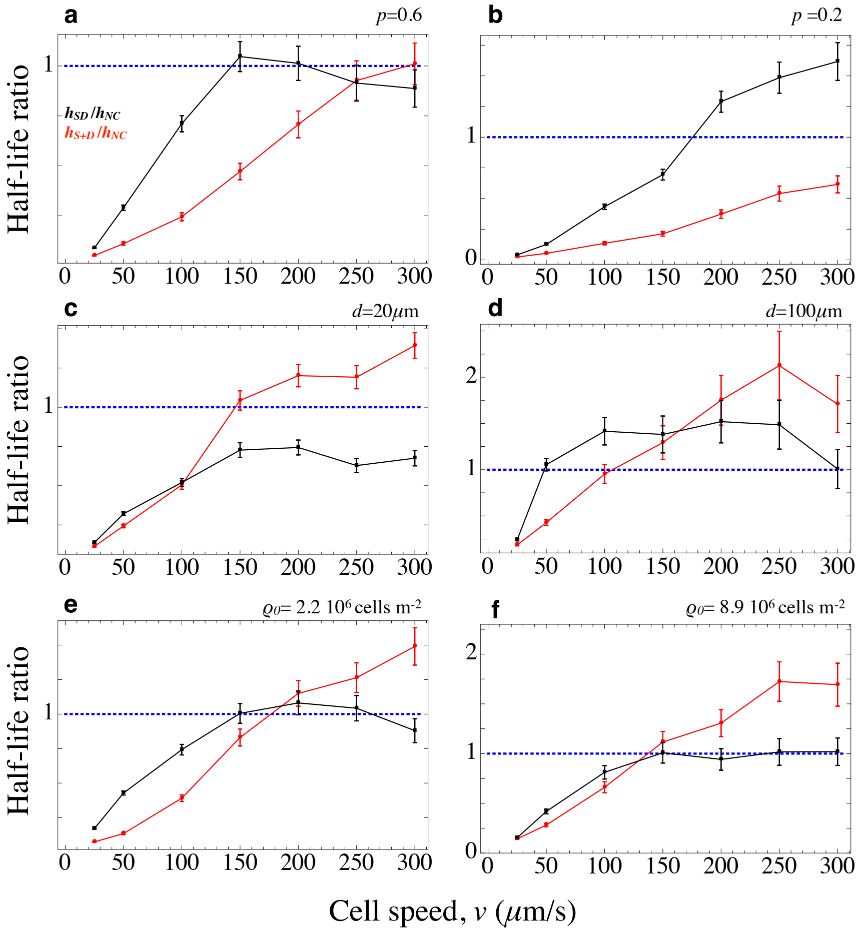

Figure S13: Mean half-life ratios with error bars (averaged over 40 simulations) for secrete-only and detect-only cells (black) compared to non-chemotactic (blue dotted)), and secrete-and-detect (black) cells, for varying cell speed. This is a repeat of Fig. 4 in the main text for different parameter sets. As a baseline we used $(\rho_0, s, u, d, \alpha, p, p_S, p_D) = (5.1\ 10^6$ cells m^{-2}, 1s^{-1}, 10^{-3}s^{-1}, 40μm, 100, 0.2, 0.2, 0.2). The ratio s/K_d is set equal to 10^{-4} and 10^{-5} for SD and S+D cells respectively.

References

[1] Johannes Taktikos, Vasily Zaburdaev, and Holger Stark. Collective dynamics of model microorganisms with chemotactic signaling. *Phys. Rev. E.*, 85, 2012.

[2] A V Hill. The possible effects of the aggregation of the molecules of haemoglobin on its dissociation curves. *J. Physiol.*, 40(4):iv–vii, 1910.

[3] Sylvain Goutelle, Michel Maurin, Florent Rougier, Xavier Barbaut, Laurent Bourguignon, Michel Ducher, and Pascal Maire. The Hill equation: A review of its capabilities in pharmacological modelling. *Fundam. Clin. Pharmacol.*, 22(6):633–648, 2008.

[4] Paul Herzmark, Kyle Campbell, Fei Wang, Kit Wong, Hana El-Samad, Alex Groisman, and Henry R Bourne. Bound attractant at the leading vs. the trailing edge determines chemotactic prowess. *Proc. Natl. Acad. Sci. U. S. A.*, 104(33):13349–54, 2007.

[5] Danny Fuller, Wen Chen, Micha Adler, Alex Groisman, Herbert Levine, Wouter-Jan Rappel, and William F Loomis. External and internal constraints on eukaryotic chemotaxis. *Proc. Natl. Acad. Sci. U. S. A.*, 107(21):9656–9, 2010.
